# Metformin and cRGDfc-Modified Nanoparticles Loaded with Curcumin for Age-Related Macular Degeneration: In Vitro Pharmacodynamics and Molecular Mechanisms

**DOI:** 10.3390/pharmaceutics18060761

**Published:** 2026-06-22

**Authors:** Juan Liu, Ziheng Wang, Yuchang Yang, Lisha Yi, Shiman Li, Jingyi Gao, Jia Zhou, Nannan Cheng, Xingbin Yin, Xiaoxv Dong, Jian Ni, Changhai Qu

**Affiliations:** School of Chinese Material Medica, Beijing University of Chinese Medicine, Beijing 102488, China

**Keywords:** age-related macular degeneration (AMD), curcumin, nanoparticle, pharmacodynamics, molecular mechanism

## Abstract

**Objectives:** This study aimed to develop curcumin nanoparticles (Cur@PCL-PEG-MF/cRGDfc) with retinal-targeting capability and to evaluate their biological effects and pharmacological mechanisms in vitro. **Methods:** After synthesis of the carrier framework, metformin (MF) and cRGDfc were conjugated to the carrier material using the carbodiimide method and Michael addition reaction, respectively. Subsequently, self-assembled nanoparticles were formed from the carrier and curcumin under specific conditions. The materials were characterized by spectroscopy, chromatography, elemental analysis, energy-dispersive spectroscopy and X-ray diffraction. The efficacy of the formulation was evaluated in two cell lines, ARPE-19 and HUVEC-T1. In addition, the pharmacological mechanism was explored using transcriptome sequencing as a complementary approach. **Key Findings:** Self-assembled nanoparticles were successfully prepared by combining the two modified carrier materials, PCL-PEG-MF and PCL-PEG-cRGDfc, with curcumin. The nanoparticles exhibited an encapsulation efficiency of 78.09%, a particle size of 162.33 nm, and a zeta potential of −23.28 mV and displayed a spherical morphology. They showed sustained release in simulated physiological conditions and stronger affinity for ARPE-19 cells under oxidative stress. Nearly 100% of the nanoparticles were internalized by the cells, which was accompanied by reduced ROS and LDH release and decreased DNA fragmentation. In addition, the nanoparticles inhibited neovascularization by reducing VEGF-A release, thereby potentially protecting the retina in macular degeneration and reducing choroidal hemorrhage. Further analyses showed that curcumin and its nanoformulations significantly reduced the expression of inflammatory factors such as IL-1β and IL-18, lowered the protein levels of Caspase-1, GSDMD-N, and NLRP3, and increased AMPK levels. **Conclusions:** Using PCL-PEG as the carrier framework, MF and cRGDfc were conjugated to construct a curcumin-loaded nanoparticle with retinal-targeting capability. This nanoparticle, characterized by a small particle size, sustained release, and targeted delivery to retinal pigment epithelium (RPE) cells under oxidative stress, alleviated oxidative stress-induced damage. Its therapeutic effect may be mediated, at least in part, by interference with the AMPK/mTOR pathway and activation of the NLRP3/Caspase-1/GSDMD pathway.

## 1. Introduction

Age-related macular degeneration (AMD) is a neurodegenerative disease and represents the third leading cause of blindness globally, following cataracts and glaucoma [1]. Wet AMD (wAMD), characterized by neovascularization and hemorrhage in the retina and choroid, accounts for only 10–15% of AMD cases but is responsible for 90% of associated blindness [2,3]. The pathological progression of wAMD involves multiple interrelated factors, including angiogenesis, immune and inflammatory responses, oxidative stress, mitochondrial and lipid metabolism dysfunction, and cellular senescence, which collectively drive disease progression [4]. The core pathological mechanism of wAMD involves retinal hypoxia or chronic inflammation, leading to overexpression of vascular endothelial growth factor A (VEGF-A) and induction of abnormal choroidal neovascularization (CNV). Current first-line treatment for wAMD consists of intravitreal injections of monoclonal anti-VEGF antibodies. However, these therapeutics exhibit short half-lives, necessitating frequent injections that increase risks of complications such as ocular infection, elevated intraocular pressure, and potential irreversible damage, including retinal detachment [3]. Owing to the presence of multiple anatomical barriers in the posterior segment—including the sclera, choroid, retinal pigment epithelium (RPE), Bruch’s membrane, and the blood–retinal barrier—drug delivery to the retina is severely constrained [5]. Consequently, intravitreal injection remains the primary administration route for retinal diseases. Thus, enhancing drug sustained-release capability and developing long-acting, targeted intravitreal formulations represent urgent challenges.

Curcumin (Cur), a compound extracted from the rhizome of *Curcuma longa* L., serves as the primary active component of turmeric [6]. It demonstrates diverse biological activities, including anti-inflammatory, antioxidant, anti-angiogenic, and neuroprotective effects. Recently, Cur has attracted significant attention in ophthalmology [7] and has emerged as a promising candidate for ophthalmic therapy [8,9,10]. However, its clinical application is limited by poor water solubility, rapid degradation under physiological conditions, low oral bioavailability, and photosensitivity [10,11]. Consequently, various novel curcumin nanoparticles have been developed for ophthalmic use, including polymer nanoparticles, liposomes [12], nanoemulsions, nanogels [13], cyclodextrin inclusion complexes [14], polymeric micelles [15], and biomimetic nanocarriers [16]. Their therapeutic efficacy has been validated in multiple ophthalmic disease models [17]. The potential of curcumin in treating AMD involves several mechanisms: its antioxidant activity may protect RPE cells from oxidative stress, delaying dry AMD progression; its anti-inflammatory activity may inhibit aberrant complement system activation and inflammasome-mediated RPE damage; and its anti-angiogenic activity may suppress VEGF and platelet-derived growth factor (PDGF) expression, thereby reducing CNV formation [18].

Efficient retinal drug delivery critically depends on the selection and modification of appropriate carriers. Nanocarrier encapsulation can isolate curcumin from the external environment, preventing degradation during storage and in vivo transport, thereby enhancing bioavailability. Polycaprolactone (PCL), a linear polyester synthesized via ε-caprolactone ring-opening polymerization, exhibits excellent encapsulation capacity for hydrophobic drugs and superior biocompatibility. PCL degrades slowly in vivo, making it suitable for long-acting, sustained-release, or implantable formulations. Its degradation generates minimal acidic byproducts, reducing inflammatory responses, which is advantageous for prolonged retinal drug release. PCL can form a diblock copolymer, PCL-PEG, with polyethylene glycol (PEG). This copolymer undergoes molecular self-assembly to form nanoparticles with sustained-release properties, constructing a nanoscale drug delivery platform [19]. The PCL-PEG diblock copolymer demonstrates high biocompatibility in the RPE region. Its biodegradation products exhibit lower acidity, potentially mitigating inflammation-related retinal cell death. Typically, the PEG terminus is capped with hydroxyl (–OH) or methyl (–OCH_3_) groups to regulate water solubility and biocompatibility. The PCL terminus usually features a hydroxyl (–OH) group; however, in this diblock copolymer, it can be further functionalized with NHS ester (–CO–NHS), maleimide (–MAL), or carboxyl (–COOH) groups for conjugation with peptides, proteins, sugars, or antibodies to enable active targeting. Metformin (MF) and cRGDfc are two key modifiers selected for carrier functionalization. Studies indicate that MF targets mitochondrial complex I [20,21], modulates the AMPK/mTOR pathway, and inhibits cellular pyroptosis [22]. Pathological alterations in mitochondrial respiratory chain complex I represent an underlying mechanism in wAMD [23]. As an AMPK/mTOR pathway modulator, MF influences the AMP/ATP ratio [24]. Additionally, its antioxidant and anti-angiogenic properties may contribute to inhibiting AMD progression. Brown et al. conducted a phase II clinical trial (NCT02684578) on geographic atrophy to evaluate MF’s safety and efficacy for ocular diseases [25]. Since AMD often involves retinal and choroidal neovascularization, and the integrin receptor αvβ3 is overexpressed on RPE cells [26], the RGD peptide—a short peptide containing the arginine–glycine–aspartic acid sequence abundant in vivo [27,28]—specifically binds αvβ integrins. Notably, a cyclic RGD peptide named cRGDfc exhibits high affinity for αvβ3 integrin, showing promise for retinal targeting [29].

## 2. Materials and Methods

### 2.1. Reagents and Chemicals

PCL-PEG, PCL-PEG-COOH, PCL-PEG-MAL, and cRGDfc were synthesized by Xi’an Ruixi Biological Technology Co., Ltd. (Xi’an, China). MF was purchased from Shanghai Aladdin Biochemical Technology Co., Ltd. (Shanghai, China). EDC·HCl, triethylamine, and DMF were obtained from Sigma-Aldrich. Tetrahydrofuran (THF) and curcumin (Cur) were purchased from Shanghai Yuan Ye Biotechnology Co., Ltd. (Shanghai, China). Pluronic F68 was acquired from Shanghai Macklin Biochemical Co., Ltd. (Shanghai, China) Polysorbate 80 was obtained from Tianjin Belsun Biotechnology Co., Ltd. (Tianjin, China). DMEM/F12 basic culture medium and fetal bovine serum were purchased from Wuhan Procell Life Science & Technology Co., Ltd. (Wuhan, China). PBS, penicillin-streptomycin mixture (P/S), 0.25% trypsin, and DMEM basic culture medium were obtained from Gibco Life Sciences. The 3% hydrogen peroxide (H_2_O_2_) was purchased from Beijing Huabo Station Bioanalytical Technology Co., Ltd. (Beijing, China). Matrigel was acquired from Xiamen Mogengel (Xiamen, China). CCK-8 detection solution was purchased from Biorigin (Beijing) Inc. (Beijng, China). The TUNEL detection kit, reactive oxygen species (ROS) detection kit, and lactate dehydrogenase (LDH) detection kit were obtained from Shanghai Beyotime Biotechnology Co., Ltd. (Shanghai, China). GAPDH Rabbit mAb (A19056) was purchased from ABclonal. VEGF-A Monoclonal antibody (66828-1-Ig), IL-18 Polyclonal antibody (10663-1-AP), mTOR Monoclonal antibody (66888-1-Ig), AMPK Alpha 1 Monoclonal antibody (66536-1-Ig), Phospho-AMPK Alpha (Thr172) Recombinant monoclonal antibody (80209-6-RR), Caspase-1/P20 Polyclonal antibody (22915-1-AP), NLRP3 Polyclonal antibody (30109-1-AP), and IL-1β Polyclonal antibody (16806-1-AP) were purchased from Abcam plc.

### 2.2. Synthesis of Carrier Materials and Preparation of Nanoparticles

PCL-PEG-MF was synthesized by dissolving 25 mg PCL-PEG-COOH and 25 mg MF in 10 mL of DMSO. Subsequently, 120 mg EDC·HCl and 90 μL triethylamine were added dropwise under ice-bath conditions. The mixture was stirred at room temperature for 48 h. Dialysis was performed using a dialysis bag (MWCO 2000) against PBS for 48 h, with the dialysate changed every 6 h. The suspension was collected and lyophilized to obtain PCL-PEG-MF as a white, odorless powder.

PCL-PEG-cRGDfc was prepared by dissolving 100 mg PCL-PEG-MAL in 3 mL of DMF. The cRGDfc peptide was added and allowed to dissolve completely. The reaction proceeded at room temperature for 12 h. The solution was dialyzed against deionized water for 24 h using a dialysis bag, with the dialysate changed every 6 h. The product was collected, lyophilized, and obtained as a white, odorless powder.

Cur@PCL-PEG was prepared by dissolving 20.0 mg curcumin in 5 mL of an organic solvent mixture (dichloromethane:ethyl acetate, 4:1 *v*/*v*). The mixture was vortexed until complete dissolution to prepare a stock solution. Then, 1 mL of the stock solution was used to dissolve 50.0 mg PCL-PEG copolymer. After vortexing, this solution served as the organic phase. Next, 2 mL of 1.5% (*w*/*v*) Pluronic F68 aqueous solution was added to the organic phase. The mixture was stirred at room temperature (1500 rpm) for 1 h. Emulsification was performed under an ice-water bath using an ultrasonic probe sonicator (600 W, 5 s pulse on/5 s off, 2 min total) until a uniform milky primary emulsion (O/W) formed. Then, 4 mL of 0.5% (*w*/*v*) Pluronic F68 solution was added to the primary emulsion. The mixture was stirred at room temperature (1500 rpm) for 6 h to evaporate organic solvents and solidify nanoparticles. The solution was transferred to centrifuge tubes and centrifuged at 4 °C (15,000 rpm) for 30 min. The supernatant was discarded. The nanoparticle pellet was resuspended in pure water, vortexed, and centrifuged under identical conditions. This washing procedure was repeated twice. Finally, the pellet was dispersed in 3 mL pure water to obtain a Cur@PCL-PEG suspension, which was stored at 4 °C in the dark.

Cur@PCL-PEG-MF/cRGDfc was prepared by dissolving 51.9 mg curcumin in 10 mL of an organic solvent mixture (dichloromethane:ethyl acetate, 4:1 *v*/*v*) to form a curcumin-containing solution. Then, 1 mL of this solution was removed. Subsequently, 40.0 mg PCL-PEG-MF copolymer and 10.0 mg PCL-PEG-cRGDfc copolymer were added to the remaining solution, and the mixture was vortexed to form the oil phase. Next, 1.85 mL of 1.5% (*w*/*v*) Pluronic F68 aqueous solution was added to the oil phase. Following a similar emulsification and solidification process as described above (stirring at room temperature, ice-water bath sonication, and solvent evaporation), Cur@PCL-PEG-MF/cRGDfc nanoparticles were obtained.

### 2.3. Characterization of Carrier Materials and Nanoparticles

Organic elemental analysis was performed using an elemental analyzer (Elementar, Langenselbold, Germany) to quantify element contents in PCL-PEG-MF, verifying MF conjugation efficiency. Samples were combusted at high temperature to convert elements into gaseous products, which were quantitatively detected by chromatography. The instrument was set to CHNS synchronous analysis mode. High-purity helium (≥99.999%) served as the carrier gas, and high-purity oxygen (≥99.995%) as the auxiliary gas. The combustion tube temperature was 1150 °C, and the reduction tube temperature was 850 °C. Dry samples were analyzed in triplicate.

Gel permeation chromatography (GPC) analysis was conducted by dissolving PCL-PEG-MAL and PCL-PEG-cRGDfc samples in THF to prepare 2 mg·mL^−1^ solutions. Samples were filtered through a 0.22 μm organic membrane before injection. GPC (Waters, Milford, MA, USA) conditions were mobile phase THF, flow rate 1.0 mL·min^−1^, column temperature 35 °C, injection volume 50 μL, and run time 15 min. Samples were injected sequentially, and chromatograms were recorded.

^1^H NMR analysis was performed by precisely weighing cRGDfc, PCL-PEG-MAL, and PCL-PEG-cRGDfc samples. These were dissolved in DMSO. ^1^H NMR spectra were acquired using an NMR spectrometer (Bruker, Mannheim, Germany) to verify successful PCL-PEG-cRGDfc conjugation.

Fourier-transform infrared (FTIR) spectroscopic analysis was conducted on Cur@PCL-PEG-MF/cRGDfc, PCL-PEG-cRGDfc, Cur, Blank@PCL-PEG, and Cur@PCL-PEG samples. Each sample was mixed with dry spectral-grade KBr powder (~1:100 mass ratio), homogenized finely, and pressed into transparent or semi-transparent pellets. Pellets were placed in the sample chamber of an FTIR spectrometer (Thermo, Waltham, MA, USA). Parameters were wavenumber range 4000–400 cm^−1^, resolution 4 cm^−1^, and 32 scans.

Energy-dispersive X-ray spectroscopy (EDS) analysis was performed by diluting Cur@PCL-PEG-MF/cRGDfc with deionized water and sonicating (100 W, 3–5 min) to ensure uniform dispersion. A 5–10 μL aliquot of the diluted solution was dropped onto a copper grid coated with an ultrathin carbon film. After standing for 2–3 min for nanoparticle adsorption, excess liquid was blotted with filter paper. The grid was air-dried at room temperature for 10–15 min. The grid was fixed on a sample holder and placed in the TEM sample chamber. Morphology was observed, and elemental surface distribution analysis was performed using an EDS spectrometer (JEOL, Tokyo, Japan) to determine C, N, O, and S distribution.

X-ray diffraction (XRD) analysis was conducted on powdered samples (Cur, Blank@PCL-PEG, Cur@PCL-PEG, Cur@PCL-PEG-MF/cRGDfc) passed through a 300-mesh sieve and evenly loaded into an X-ray diffractometer (Bruker, Germany). Conditions were Cu-Kα radiation, diffraction angle (2θ) range 3–60°, scan speed 3°·min^−1^, tube voltage 40 kV, and tube current 40 mA. All tests were performed at room temperature.

The particle size, polydispersity index (PDI), and zeta potential of nanoparticles were measured using a laser particle analyzer (Malvern, Malvern, UK). Morphology was observed by transmission electron microscopy (TEM, JEOL, Japan). Briefly, nanoparticle suspensions were appropriately diluted and sonicated (100 W, 3–5 min) for uniform dispersion. For particle size and zeta potential measurements, a 1 mL sample was placed in the analyzer with the following parameters: dispersant pure water, temperature 25 °C, and equilibration time 120 s. Each sample was measured in triplicate. For TEM, a 10 μL sample solution was applied to a copper grid coated with an ultrathin carbon film. Then, 2.0% (*w*/*v*) phosphotungstic acid (PTA) solution was added for negative staining. The grid was examined by TEM to observe nanoparticle morphology and capture images.

### 2.4. Encapsulation Efficiency (EE) and Drug Loading (DL)

Curcumin content was quantified by high-performance liquid chromatography (HPLC) under the following conditions: Diamonsil Plus C18 column (250 mm × 4.6 mm, 5 μm); mobile phase acetonitrile–4% glacial acetic acid solution (48:52); flow rate 1.0 mL·min^−1^; detection wavelength 430 nm; column temperature 30 °C; and injection volume 10 μL.

The Cur@PCL-PEG suspension was centrifuged at 15,000 r·min^−1^ for 30 min. The precipitate was washed twice with pure water. All supernatants and washing solutions were combined and diluted to volume with methanol. The solution was mixed well and filtered through a 0.45 μm membrane. The filtrate was analyzed as the test sample. The curcumin peak area was recorded and substituted into Equations (1) and (2) to calculate EE and DL.

DL% = (Mass of encapsulated drug/Mass of nanoparticles) × 100%(1)

EE% = (Mass of encapsulated drug/Initial drug mass) × 100%(2)

### 2.5. Stability

The prepared Cur@PCL-PEG and Cur@PCL-PEG-MF/cRGDfc solutions were stored at 4 °C. Three replicates per formulation were prepared. Particle size was monitored daily for 7 days using a laser particle size analyzer to assess stability.

### 2.6. In Vitro Release of Nanoparticles

In vitro release was evaluated using the shaker method [30]. Briefly, triplicate samples of Cur@PCL-PEG and Cur@PCL-PEG-MF/cRGDfc (each containing 2.0 mg curcumin) were placed in 25 mL PBS (pH 7.4) release medium containing 1% polysorbate-80. The addition of 1% polysorbate-80 significantly improved the solubility of curcumin in the release medium. Combined with the sufficient medium volume and regular replenishment of fresh medium at each sampling, sink conditions were maintained throughout the experiment. Samples were incubated in a 37 °C shaking incubator at 100 r·min^−1^ in the dark. At predetermined time points (1, 2, 4, 8, 12, 24, 48, 72, 96, 120, 144 h), 1.0 mL of the release medium was sampled. The sampled medium was mixed well and immediately replaced with an equal volume of fresh medium to maintain sink conditions. Samples were centrifuged at 10,000 r·min^−1^ for 10 min. The supernatant was aspirated, filtered through a 0.45 μm membrane, and analyzed by HPLC to determine curcumin content. Cumulative release rates were calculated, and release curves were plotted.

### 2.7. Cell Culture and Treatment

The STR-authenticated ARPE-19 cells (Cat. No. CL-0026) and PUMC-HUVEC-T1 cells (Cat. No. CL-0675) were both purchased from Procell Life Science & Technology Co., Ltd. (Wuhan, China). All cells were cultured under standard sterile conditions. ARPE-19 cells were cultured in DMEM/F12 supplemented with 10% FBS and 1% P/S at 37 °C in a humidified 5% CO_2_ atmosphere. HUVEC-T1 cells were cultured in DMEM with 10% FBS, 1% P/S, and 1% non-essential amino acids (NEAAs) under the same conditions. Cells at 80–90% confluence were passaged for experiments. Groups were the Normal group (no intervention); Model group (culture medium containing 3% H_2_O_2_); and Drug intervention groups (media containing specified concentrations of Cur@PCL-PEG, Cur@PCL-PEG, and Cur@PCL-PEG-MF/cRGDfc, respectively). After treatment, cell viability assays and other experiments were conducted to evaluate treatment effects.

### 2.8. Cell Viability Assay and Morphology Examination

For drug effects on cell damage and protection, cells were seeded in 96-well plates at 5 × 10^3^ cells/well. Viability was assessed using the Cell Counting Kit-8 (CCK-8). Cells were incubated in serum-free medium containing 10% CCK-8 for 1–2 h, and absorbance was measured at 450 nm using a microplate reader (BioTek, Miami, FL, USA). Cell viability (%) was calculated as [(OD_experimental − OD_blank)/(OD_normal − OD_blank)] × 100%. After 24 h stimulation with drug solutions in serum-free medium, the safe concentration of curcumin and its nanoformulations for ARPE-19, the damaging concentration of H_2_O_2_ for ARPE-19, and the cytotoxic effects on HUVEC cells were determined. For protection studies, cells were pretreated with different concentrations for 24 h. After discarding the drug-containing medium, cells were stimulated with H_2_O_2_ for 3 h. Cell morphology was observed using a fluorescence microscope (Olympus, Tokyo, Japan).

### 2.9. Lactate Dehydrogenase (LDH) Release Assay

Using the aforementioned cell grouping, after 24 h intervention with the corresponding reagents, cells were stimulated with 400 μM H_2_O_2_ for 3 h to establish an oxidative stress model. The cell culture supernatant was collected, and optical density (OD) was measured at 490 nm according to the LDH kit instructions. LDH release was compared. Experiments were independently repeated three times.

### 2.10. ROS Measurement

Using the same cell grouping and treatment methods, the DCFH-DA probe was diluted as per the ROS detection kit instructions. Cells were incubated at 37 °C for 20 min. ROS levels were observed using a fluorescence inverted microscope (Olympus, Japan) or quantitatively analyzed by flow cytometry (BD, Franklin Lakes, NJ, USA) after cell collection.

### 2.11. Cellular Uptake

A nanoparticle-containing medium was added to both normal cultured cells and H_2_O_2_-induced oxidative stress model cells. After 4 h incubation, cells were harvested and analyzed by flow cytometry (BD, Franklin Lakes, NJ, USA) to assess nanoparticle uptake.

### 2.12. Cell Scratch Assay

HUVEC cells in the logarithmic growth phase were uniformly seeded into 6-well plates. Vertical scratches parallel to plate reference lines were made using a 200 μL pipette tip. Plates were washed thrice with PBS to remove detached cells, then replaced with serum-free medium. Images were captured using a fluorescence inverted microscope (4× objective). The drug-containing medium was added according to grouping, and images were taken 12 h post-scratch. The scratch area was quantified using ImageJ (v1.53a, National Institutes of Health, Bethesda, MD, USA) software. The cell migration rate (%) was calculated as (Initial scratch area − Scratch area at time t)/Initial scratch area × 100%.

### 2.13. Extracellular Tube Formation Assay

Matrigel was used as a three-dimensional culture matrix. A 20 μL aliquot of Matrigel was added to each well of a 24-well plate. The plate was incubated at 37 °C for 60 min for gel solidification. For each group, 250 μL of cell suspension (1.5 × 10^5^ cells/well) and 250 μL of the drug-containing medium (Cur, Cur@PCL-PEG, or Cur@PCL-PEG-MF/cRGDfc groups) were added per well. Triplicates were prepared per group. The plate was incubated at 37 °C, 5% CO_2_. Images were captured using a fluorescence inverted microscope, and total tube length was quantified.

### 2.14. TUNEL Staining

A high-content cell imaging analysis system (BD, USA) was used to evaluate the effects of different curcumin formulations on DNA fragmentation in ARPE-19 cells under oxidative stress. The same cell grouping was adopted. Staining was performed according to the TUNEL kit instructions. Images were observed and collected using the high-content cell imaging analyzer.

### 2.15. Transcriptomics Analysis

Using the same cell grouping and treatment, total RNA was extracted from each group using TRIzol reagent. RNA concentration and purity were detected using the NanoDrop ND-1000 (Thermo Fisher Scientific, Waltham, MA, USA), and integrity was assessed by the Bioanalyzer 2100 (Agilent Technologies, Santa Clara, CA, USA). Samples with a concentration > 50 ng·μL^−1^, RNA integrity number (RIN) > 7.0, and total amount > 1 μg were selected. mRNA was enriched using oligo(dT) magnetic beads, and libraries were constructed. After quality control, paired-end sequencing was performed on the Illumina NovaSeq 6000 platform. Raw data were quality-controlled to obtain high-quality sequences. Genome alignment, gene expression quantification, gene set enrichment analysis, differential gene expression analysis, and enrichment analysis were conducted.

### 2.16. Western Blot

Using the same cell grouping and treatment, the culture medium was discarded, and pre-cooled RIPA lysis buffer (containing 1% protease inhibitor and 1% phosphatase inhibitor) was added to the cells. The cells were lysed on ice for 30 min. Samples were centrifuged at 12,000 r·min^−1^, 4 °C, for 10 min. The supernatant was collected as total protein. The protein concentration was determined by BCA assay. Loading buffer was added, and the protein amount was adjusted to 25 μg/10 μL per group. Samples were heated at 95 °C for 10 min, followed by electrophoresis and membrane transfer. Antibodies were incubated according to manufacturer-specified dilution ratios.

### 2.17. Data Analysis

Statistical analyses were performed using GraphPad Prism 9.5 (GraphPad Software, Boston, MA, USA). Data are presented as mean ± standard deviation (SD) from at least three independent experiments. One-way analysis of variance (ANOVA) was used for multiple-group comparisons, followed by Tukey’s multiple comparisons test for pairwise comparisons. Statistical significance was defined as *p* < 0.05. Image analysis was conducted using ImageJ 1.53a software (National Institutes of Health, Bethesda, MD, USA) with the Angio Analyzer plugin.

## 3. Results

### 3.1. Amide Bond Coupling for PCL-PEG-MF Synthesis

**Figure 1:**
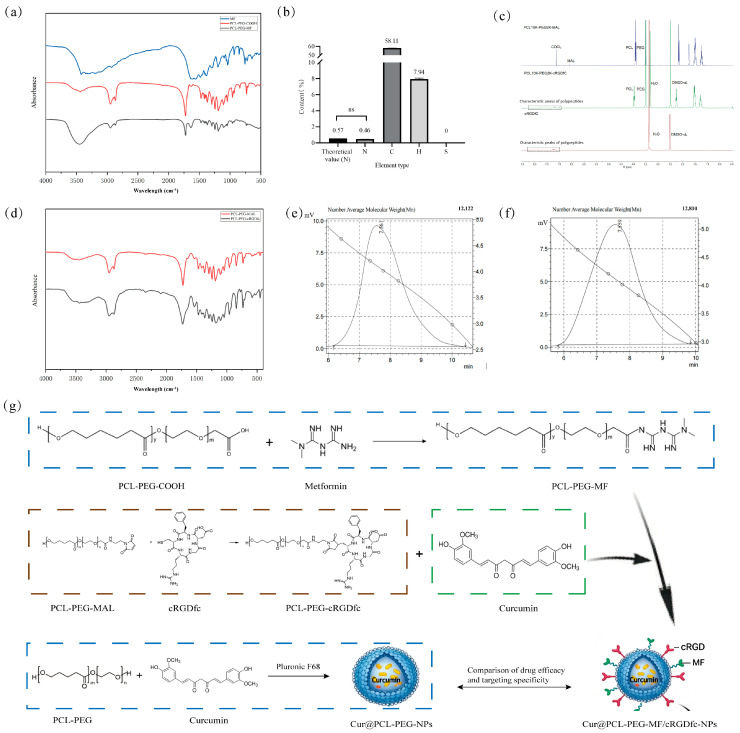
Carrier synthesis and characterization (**a**) FTIR spectra of MF, PCL-PEG-COOH, and PCL-PEG-MF; (**b**) elemental analysis of PCL-PEG-MF; (**c**) ^1^H NMR spectra of cRGDfc, PCL-PEG-MAL, and PCL-PEG-cRGDfc; (**d**) FTIR spectra of PCL-PEG-MAL and PCL-PEG-cRGDfc; (**e**) GPC chromatogram of PCL-PEG-MAL; (**f**) GPC chromatogram of PCL-PEG-cRGDfc); (**g**) Schematic diagram of the preparation process of nanoparticles.

FTIR analysis was employed to identify functional groups and infer the conjugation mode [31]. In the MF structure, the N–H stretching vibration of the guanidine moiety displayed a broad peak due to hydrogen bonding (Figure 1a). The C=N stretching vibration at 1620–1650 cm^−1^ is characteristic. In PCL-PEG-COOH, the ester group and terminal carboxyl C=O stretching vibrations produced a strong, sharp peak at 1720–1750 cm^−1^. The C–O–C stretching of PEG formed a strong peak at 1100–1150 cm^−1^, overlapping with the C–O peak of PCL (1100–1200 cm^−1^) (Figure 1a). After reaction, the sharp C=O peak at 1720–1750 cm^−1^ weakened due to amide formation. The N–H peak of metformin’s primary amine (3300–3500 cm^−1^) also diminished, indicating amino group consumption. Concurrently, characteristic amide bond peaks appeared: a strong peak at 1630–1680 cm^−1^ (amide I band, C=O stretch) and a medium-strong peak near 1550 cm^−1^ (amide II band, N–H bend + C–N stretch coupling). Carboxyl consumption led to significant weakening of the O–H peak at 3000 cm^−1^, while N–H stretching broadened due to hydrogen bonding, forming a broad peak at 3300–3500 cm^−1^.

Based on the reaction mechanism, the amino group in MF reacts with the terminal carboxyl of PCL-PEG-COOH. Assuming a 1:1 molar ratio, the theoretical nitrogen content of PCL-PEG-MF was calculated as 0.57%. Organic elemental analysis of the product yielded an average nitrogen content of 0.46%. Statistical analysis showed no significant difference between measured and theoretical values (*p* > 0.05) (Figure 1b).

### 3.2. Thiol-Maleimide Conjugation for PCL-PEG-cRGDfc Synthesis

A strong absorption peak around 1720 cm^−1^ (Figure 1d) corresponds to the C=O stretching vibration of the PCL ester. The strong peak around 1100 cm^−1^ is attributed to C–O–C stretching of the PEG segment. Both spectra retained their characteristic peaks, indicating integrity of the PEG and PCL chains. Maleimide (MAL) theoretically exhibits characteristic absorption at ~1630–1650 cm^−1^ (C=C double bond stretch) and ~1700 cm^−1^ (imide C=O stretch), with the latter overlapping with the PCL ester carbonyl peak. After reaction, the C=C double bond peak at ~1630 cm^−1^ disappeared, indicating MAL double bond consumption via the Michael addition reaction [32]. Concurrently, absorption intensity increased at ~1650 cm^−1^ (amide I band) and ~1550 cm^−1^ (amide II band), characteristic of the cRGDfc peptide amide bonds, confirming peptide introduction. Moreover, the product’s absorption at 3300 cm^−1^ broadened and intensified, corresponding to N–H stretching in the peptide chain, further verifying peptide presence.

^1^H NMR results further confirmed conjugation (Figure 1c). MAL displayed a distinct singlet at 6.7 ppm, corresponding to the equivalent double bonds (–CH=CH–) in the maleimide ring, serving as a key reaction marker. This double bond peak disappeared in the product, indicating maleimide double bond consumption. Simultaneously, characteristic peaks of the cyclic peptide cRGDfc emerged. New multiplets appeared in the low-field region (7–8.5 ppm), matching the “peptide characteristic peak” position of pure cRGDfc (red spectrum) and attributable to aromatic hydrogens (from phenylalanine/tyrosine) and amide NH hydrogens. Main chain signals remained intact post-reaction: PCL’s –OCH_2_– peak at ~4.0 ppm, PEG’s –OCH_2_CH_2_– at ~3.6 ppm, and PCL methylene’s at 1.3–1.7 ppm. Peak shapes and relative proportions remained stable, indicating no polymer main chain damage during reaction.

In GPC analysis, larger molecules have larger hydrodynamic volumes and shorter retention times due to greater column exclusion. The elution time of PCL-PEG-cRGDfc was 7.559 min, earlier than PCL-PEG-MAL (7.561 min) (Figure 1f), directly indicating increased molecular weight. Furthermore, GPC showed the average molecular weight of PCL-PEG-MAL as 12,122 and PCL-PEG-cRGDfc as 12,810, with a molecular weight increase of 688, confirming successful cRGDfc conjugation.

### 3.3. Nanoparticles Exhibit Stability and Sustained-Release Properties

**Figure 2:**
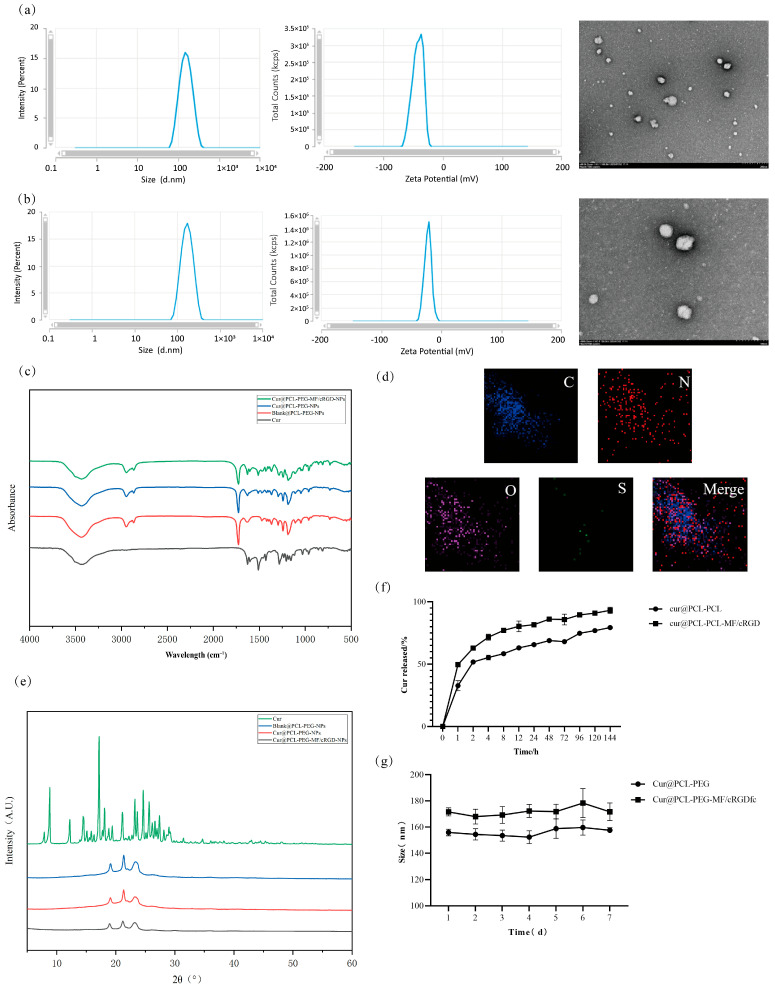
Structural analysis and characteristics of two types of nanoparticles (**a**) Particle size, zeta potential and TEM results of Cur@PCL-PEG; (**b**) particle size, zeta potential and TEM results of Cur@PCL-PEG-MF/cRGDfc-NPs; (**c**) FTIR spectra of Cur, PCL-PEG, Cur@PCL-PEG, and Cur@PCL-PEG-MF/cRGDfc; (**d**) EDS analysis of Cur@PCL-PEG-MF/cRGDfc-NPs; (**e**) XRD patterns of Cur, PCL-PEG, Cur@PCL-PEG, and Cur@PCL-PEG-MF/cRGDfc; (**f**) in vitro release profiles of the two drug-loaded nanoparticles; (**g**) particle size variation curves of both nanoparticle types during storage at 4 °C).

Curcumin-loaded nanoparticles were fabricated using these two materials (Figure 1g). The average particle size of Cur@PCL-PEG measured (149.60 ± 0.44) nm, with a polydispersity index (PDI) of 0.18 ± 0.03 and a zeta potential of (−41.29 ± 0.91) mV (Figure 2a). For Cur@PCL-PEG-MF/cRGDfc, the average particle size was (162.33 ± 2.06) nm, with a PDI of 0.21 ± 0.01 and a zeta potential of (−23.28 ± 0.49) mV (Figure 2b). Both nanoparticle types displayed regular morphology, appearing as well-defined spherical or near-spherical structures. The encapsulation efficiency (EE) and drug loading (DL) of Cur@PCL-PEG-MF/cRGDfc-NPs were (78.09 ± 0.16)% and (7.50 ± 0.01)%, respectively. The non-targeted Cur@PCL-PEG-NPs exhibited a slightly lower EE of (73.69 ± 0.86)% and DL of (7.11 ± 0.08)%.

When stored at 4 °C for 7 days, both Cur@PCL-PEG and Cur@PCL-PEG-MF/cRGDfc maintained stable particle sizes, indicating excellent storage stability (Figure 2g). Both unmodified and dual-targeted (MF and cRGDfc) modified curcumin nanoparticles demonstrated pronounced sustained-release characteristics (Figure 2f). The initial 1 h represented a rapid release phase, during which free drug molecules and the surface-adsorbed drug rapidly diffused into the aqueous medium. Subsequently, the release rate progressively decelerated, fully manifesting the sustained-release property inherent to PCL. The drug encapsulated within the nanoparticle core exhibited an extremely slow release profile. For Cur@PCL-PEG-MF/cRGDfc, the cumulative release from 2 h to 144 h reached only approximately 30%, with an average hourly release rate of about 0.2%. These remarkably slow release kinetics aligned with the design objective of long-term sustained drug delivery. Furthermore, the total cumulative release attained 93.11% at 144 h, indicating near-complete drug release from the nanoparticles over time.

### 3.4. Structural Analysis of Drug-Loaded and Targeted-Modified Nanoparticles

Energy-dispersive X-ray spectroscopy (EDS) characterization revealed the elemental composition and spatial distribution within the nanoparticles. The results demonstrated the presence of four characteristic elements: C, N, O, and S (Figure 2d). The C element signal showed high-density aggregation predominantly localized in the nanoparticle core region, while N, O, and S element signals were relatively dispersed, being uniformly distributed throughout the nanoparticle outer contour. This elemental distribution pattern strongly correlated with the expected structure obtained via emulsification-solvent evaporation preparation: During nanoparticle formation, the amphiphilic block copolymer PCL-PEG self-assembles, with hydrophobic PCL segments folding inward to form the core, effectively encapsulating curcumin (primarily contributing C element signal); hydrophilic PEG segments extend outward to form the shell, with terminal chemical modifications connecting dual-targeting molecules MF (containing N and O elements) and cRGDfc (containing N, O, and S elements), thereby enriching characteristic N, O, and S signals in the nanoparticle outer layer. These findings confirm that MF and cRGDfc are successfully modified on the nanoparticle surface, facilitating optimal targeting functionality.

The FTIR spectra of Cur@PCL-PEG and Cur@PCL-PEG-MF/cRGDfc showed fundamental consistency with Blank@PCL-PEG in major characteristic absorption peaks, while characteristic peaks corresponding to typical aromatic ring skeleton vibrations in raw curcumin material were absent from drug-loaded nanoparticle spectra (Figure 2c). This indicates that curcumin is not merely physically adsorbed on nanoparticle surfaces but is effectively encapsulated within the PCL-PEG polymer matrix.

Crystalline substances typically exhibit sharp characteristic diffraction peaks in XRD patterns corresponding to specific lattice plane reflections, while amorphous materials display broad diffuse bands due to structural disorder [32] (Figure 2e). Curcumin showed intense diffraction peaks at 2θ angles of 8.8°, 17.2°, 24.6°, and 25.6°, indicating its highly ordered crystalline state. In contrast, XRD spectra of Cur@PCL-PEG and Cur@PCL-PEG-MF/cRGDfc exhibited only characteristic crystalline peaks of carrier materials PEG and PCL at 19.2°, 21.3°, and 23.2°, with the complete disappearance of original curcumin diffraction peaks. These results confirm successful curcumin encapsulation within the PCL-PEG nanoparticle matrix in a highly dispersed amorphous or molecular state. Furthermore, MF and cRGDfc modification introduced no new diffraction peaks, suggesting the surface modification procedure did not alter the drug encapsulation structure or dispersion state.

### 3.5. Nanoparticles Significantly Ameliorate H_2_O_2_-Induced ARPE-19 Damage

**Figure 3:**
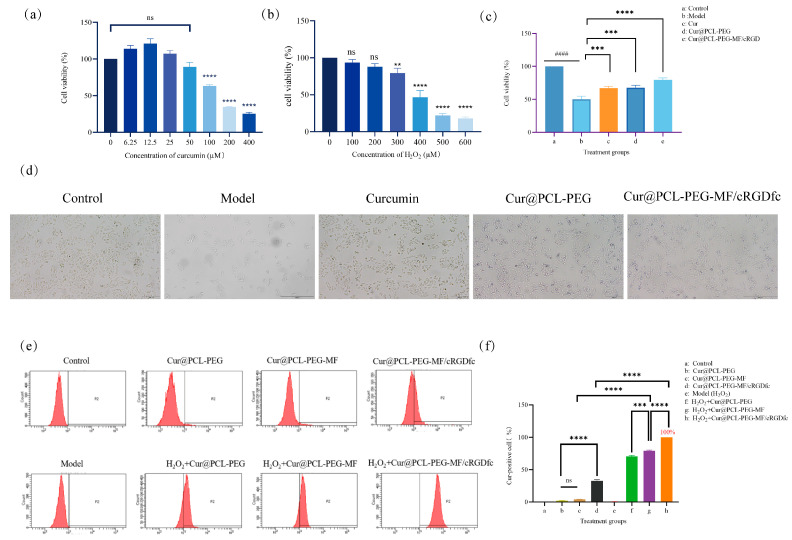
Nanoparticles demonstrate enhanced cellular uptake in ARPE-19 cells and ameliorate H_2_O_2_-induced damage (**a**) Cell viability of ARPE-19 cells treated with increasing concentrations of curcumin (0–400 μM); (**b**) cell viability of ARPE-19 cells treated with increasing concentrations of H_2_O_2_ (0–600 μM); (**c**) protective effects of curcumin and its formulations against H_2_O_2_-induced cell damage; (**d**) morphological observations of ARPE-19 cells under different treatment conditions; (**e**,**f**) cellular uptake of curcumin and its nano-formulations in normal and oxidative stress-induced ARPE-19 cells. All data are expressed as mean ± standard deviation (SD) from at least three independent experiments. Statistical significance was assessed using one-way analysis of variance (ANOVA) followed by Tukey’s multiple comparisons test. For panels (**a**,**b**), all pairwise comparisons were made relative to the 0 μM untreated control group of each experiment. Significance levels: ** *p* < 0.01, *** *p* < 0.001, and **** *p* < 0.0001 vs. control; ns, not significant (*p* ≥ 0.05 vs. control), #### *p* < 0.0001 vs. Model.

An oxidative damage model was established in ARPE-19 cells using H_2_O_2_ (Figure 3a), with cellular damage exhibiting concentration-dependent effects within the 0–600 μM range. A 400 μM H_2_O_2_ concentration was selected for model establishment. Cell proliferation assays demonstrated a curcumin safety concentration below 50 μM for retinal pigment epithelium (RPE) cells (Figure 3b), leading to the selection of 25 μM curcumin for pharmacological evaluation. Subsequent comparison of the antioxidative damage effects among curcumin nano-formulations within safe concentration ranges revealed that Cur, Cur@PCL-PEG, and Cur@PCL-PEG-MF/cRGDfc all ameliorated H_2_O_2_-induced ARPE-19 damage to varying degrees, with targeted-modified Cur@PCL-PEG-MF/cRGDfc showing superior efficacy (Figure 3c). Microscopic observation revealed H_2_O_2_-treated ARPE-19 cells exhibited typical pyroptosis characteristics, including membrane rupture and cytoplasmic content leakage. Drug treatment significantly improved morphological damage across all treatment groups, restoring membrane integrity, alleviating rupture phenomena, and effectively reversing cell number reduction trends. Microscopic examination confirmed all three drug formulations improved H_2_O_2_-induced morphological alterations (Figure 3d).

### 3.6. Nanoparticles Exhibit Retinal Targeting Properties, Particularly Under Oxidative Stress Conditions

To elucidate the superior performance of targeted nano-formulations over drug solutions and unmodified formulations, we hypothesized that nanoparticles preferentially target oxidative stress-experiencing cells, facilitating enhanced cellular uptake and efficacy. Cellular uptake experiments demonstrated flow cytometry detection of cRGDfc/MF co-modified, MF-only modified, and unmodified nanoparticle uptake by ARPE-19 cells under normal and model conditions (Figure 3e,f). In normal cells, the Cur@PCL-PEG-MF/cRGDfc group showed significantly increased positive cell proportion compared to unmodified nanoparticles (*p* < 0.0001), while the MF-only modified group (Cur@PCL-PEG-MF) showed no statistical difference. These results clearly demonstrate Cur@PCL-PEG-MF/cRGDfc’s higher affinity for ARPE-19 cells, primarily attributable to cRGDfc modification. In the H_2_O_2_-stimulated ARPE-19 damage model, all nanoparticle groups exhibited significantly enhanced uptake compared to non-modeled normal cells. Flow cytometry revealed approximately 100% curcumin-positive cells in the Cur@PCL-PEG-MF/cRGDfc group, significantly exceeding the Cur@PCL-PEG-MF and Cur@PCL-PEG groups (Figure 3f). These findings further confirm that cRGDfc modification significantly enhances curcumin nanoparticle affinity for ARPE-19 cells, particularly under oxidative stress conditions where modified nanoparticles are more effectively internalized, facilitating pharmacological activity.

### 3.7. Nanoparticles Reduce LDH Release, Decrease ROS Levels, and Alleviate DNA Fragmentation via Inhibiting Cellular Pyroptosis

**Figure 4:**
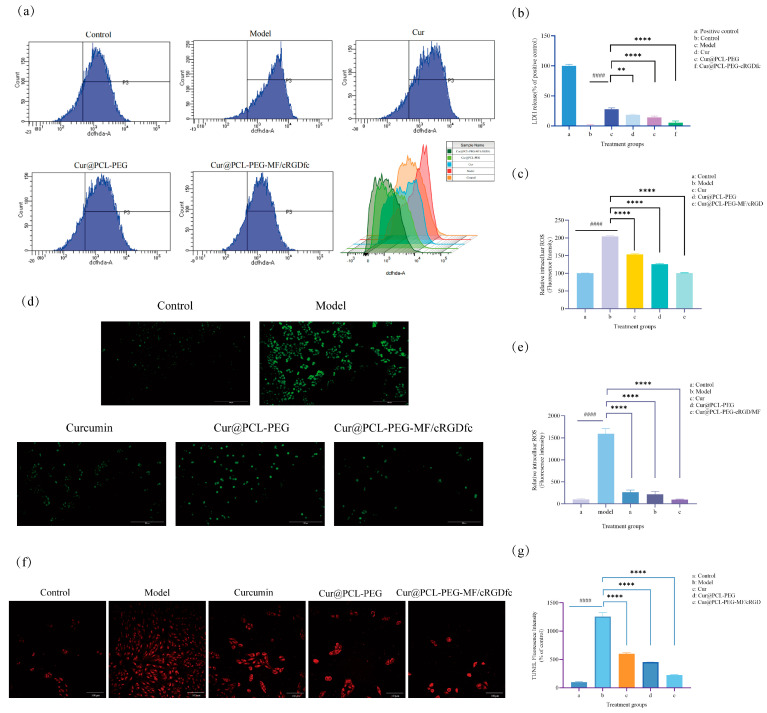
Curcumin and its nano-formulations protect ARPE-19 cells from oxidative stress by reducing ROS, LDH release, and DNA damage (**a**,**c**) Flow cytometric detection of ROS release and statistical analysis; (**b**) LDH release results; (**d**,**e**) fluorescence microscopic observation of ROS and statistical analysis; (**f**,**g**) TUNEL staining and statistical analysis). Significance levels: ** *p* < 0.01, **** *p* < 0.0001 vs. control; #### *p* < 0.0001 vs. Model.

Under pyroptotic conditions, cellular membrane integrity is compromised, leading to LDH leakage into culture medium, making supernatant LDH release a reliable pyroptosis biomarker. After 3 h H_2_O_2_ treatment, ARPE-19 cells showed significantly increased LDH release (*p* < 0.0001), confirming successful oxidative stress-induced membrane disruption. This is consistent with previous reports [33]. Drug interventions resulted in varying degrees of LDH release reduction, with the Cur@PCL-PEG-MF/cRGDfc group showing the lowest LDH release and most significant improvement (*p* < 0.0001), indicating the effective protection of membrane integrity against H_2_O_2_-induced damage (Figure 4b). Additionally, H_2_O_2_ elevated intracellular reactive oxygen species (ROS) levels, while curcumin and its nano-formulations reduced ROS levels, with Cur@PCL-PEG-MF/cRGDfc showing the most pronounced effects, consistent with flow cytometry and fluorescence microscopy observations (Figure 4a,c,e). Cellular pyroptosis typically involves DNA fragmentation, observable via TUNEL staining. Curcumin and its nano-formulations effectively ameliorated H_2_O_2_-induced DNA fragmentation (Figure 4f,g). These results collectively demonstrate that curcumin and its nano-formulations exert protective effects by mitigating pyroptosis.

**Figure 5:**
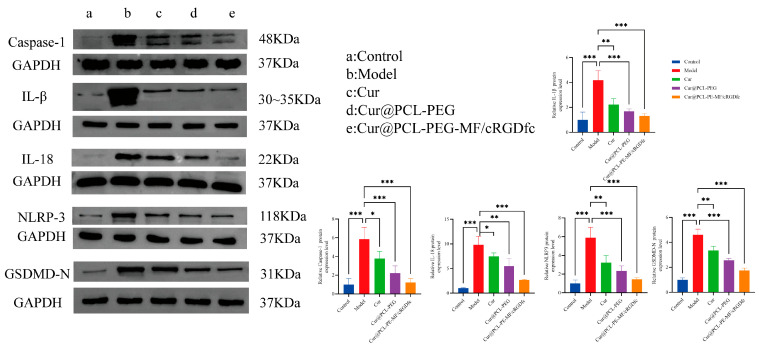
Curcumin and its nano-formulations regulate the expression of proteins related to the NLRP3/Caspase-1/GSDMD pyroptosis pathway. Representative Western blots (**left panels**) show proteins from three independent biological replicates, with GAPDH as the loading control. Quantitative analysis (**right panels**) presents mean ± SD of band intensities normalized to GAPDH (n = 3). Statistical significance was determined by one-way ANOVA followed by Tukey’s post hoc test. Significance levels: * *p* < 0.05, ** *p* < 0.01, *** *p* < 0.001 vs. Model.

To further validate the involvement of pyroptosis, we examined key proteins in the NLRP3/Caspase-1/GSDMD pathway (Figure 5). Consistent with the phenotypic results, the model group showed marked upregulation of NLRP3, cleaved Caspase-1, GSDMD-N, IL-1β, and IL-18. Cur@PCL-PEG-MF/cRGDfc treatment significantly downregulated these pyroptosis-related proteins, confirming its inhibitory effect on the inflammatory cascade.

Collectively, these findings from both functional assays and Western blotting demonstrate that Cur@PCL-PEG-MF/cRGDfc protects ARPE-19 cells from oxidative stress by suppressing NLRP3/Caspase-1/GSDMD-mediated pyroptosis.

### 3.8. Nanoparticles Inhibit Angiogenesis

**Figure 6:**
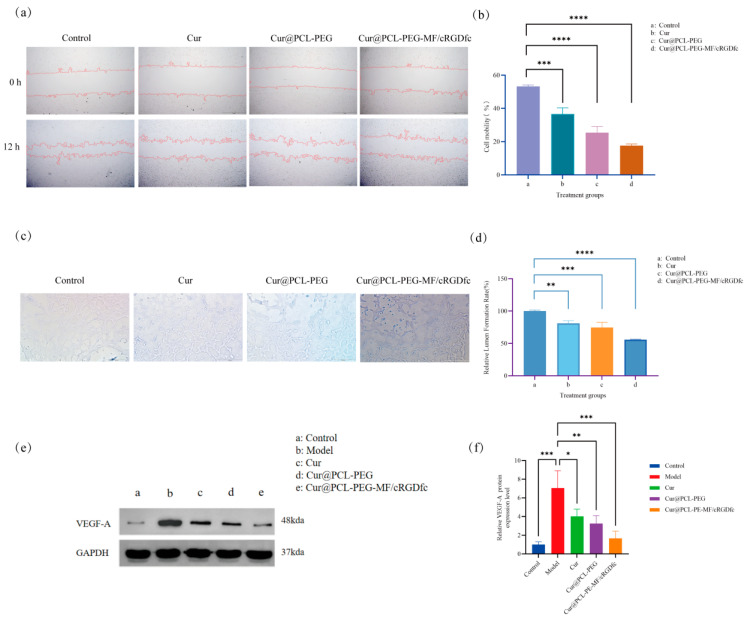
Curcumin and its nano-formulations inhibit angiogenesis (**a**,**b**) HUVEC scratch assay and statistical analysis; (**c**,**d**) HUVEC tube formation assay and statistical analysis; (**e**) representative Western blots from three independent biological replicates. (**f**) quantitative analysis of VEGF-A band intensities normalized to GAPDH. Bars represent mean ± SD of three independent experiments. Statistical significance was determined by one-way ANOVA followed by Tukey’s post hoc test. Significance levels: * *p* < 0.05, ** *p* < 0.01, *** *p* < 0.001, **** *p* < 0.0001 vs. Model.

Scratch assays evaluated HUVEC migration capacity. After 12 h interventions, Cur, Cur@PCL-PEG, and Cur@PCL-PEG-MF/cRGDfc groups all showed significantly reduced migration rates compared to controls (*p* < 0.001), with Cur@PCL-PEG-MF/cRGDfc exhibiting the most potent inhibition (*p* < 0.0001) (Figure 6a,b). Matrigel tube formation assays assessed HUVEC angiogenic capability, revealing significant anti-angiogenic effects across all treatment groups (*p* < 0.01), with Cur@PCL-PEG-MF/cRGDfc showing the strongest inhibition (*p* < 0.0001) (Figure 6c,d). These findings indicate curcumin and its nano-formulations effectively suppress HUVEC migration and tube formation, potentially inhibiting retinal-choroidal angiogenesis in AMD. Since neovascularization often results from vascular endothelial growth factor A (VEGF-A) overexpression, we examined VEGF-A secretion by ARPE-19 cells under oxidative stress. Curcumin and its nano-formulations significantly ameliorated H_2_O_2_-induced VEGF-A overexpression (Figure 6e,f).

### 3.9. Curcumin and Its Nano-Formulations Protect Cells via AMPK-mTOR Pathway Activation and NLRP3/Caspase-Driven Inflammation Inhibition

**Figure 7:**
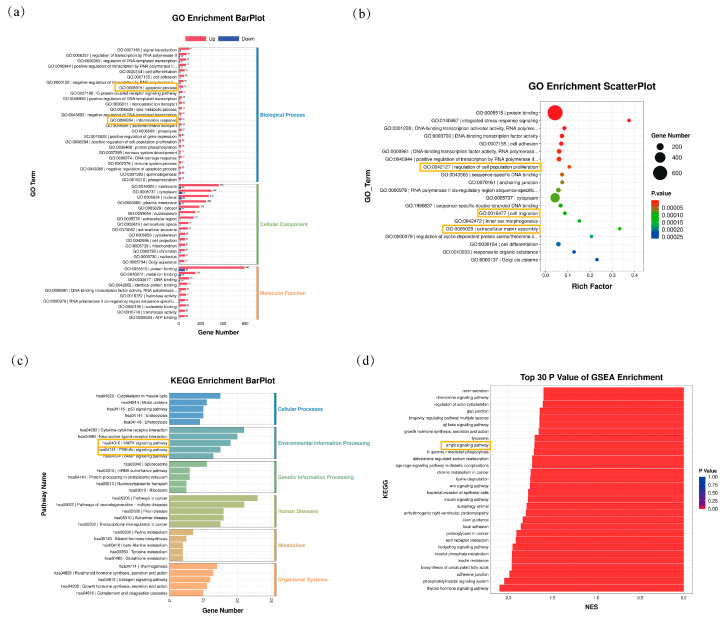
Transcriptome sequencing and pathway enrichment analysis of H_2_O_2_-treated cells. (**a**) Bar chart of GO functional enrichment analysis for differentially expressed genes. (**b**) Bubble plot of GO functional enrichment analysis for differentially expressed genes. (**c**) Bar chart of KEGG pathway enrichment analysis for differentially expressed genes. (**d**) Bar chart of top 30 pathways ranked by P value in GSEA enrichment analysis.

**Figure 8:**
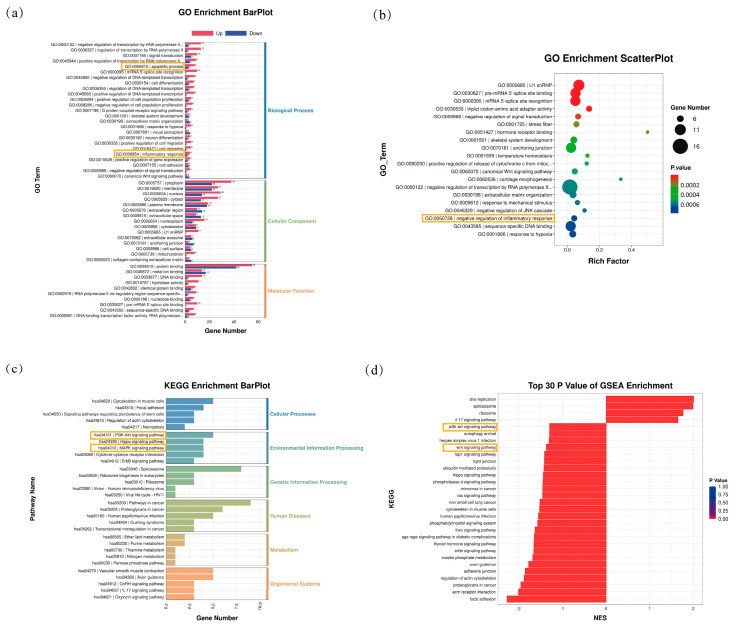
Transcriptome sequencing and pathway enrichment analysis of curcumin-treated cells. (**a**) Bar chart of GO functional enrichment analysis for differentially expressed genes. (**b**) Bubble plot of GO functional enrichment analysis for differentially expressed genes. (**c**) Bar chart of KEGG pathway enrichment analysis for differentially expressed genes. (**d**) Bar chart of top 30 pathways ranked by P value in GSEA enrichment analysis.

Transcriptomic analysis elucidated curcumin’s mechanism of action. Compared to controls, H_2_O_2_ treatment identified 2528 differentially expressed genes (90 downregulated, 1628 upregulated). GO enrichment analysis revealed significant enrichment in apoptosis and inflammation-related biological processes (Figure 6a,b). KEGG pathway analysis confirmed H_2_O_2_ activation of apoptosis and inflammation pathways (Figure 6c). Gene set enrichment analysis indicated H_2_O_2_ significantly inhibited the AMPK signaling pathway (Figure 6d), suggesting energy metabolism dysregulation involvement in oxidative injury. Curcumin intervention identified 233 differentially expressed genes versus the H_2_O_2_ group (79 downregulated, 154 upregulated). GO and KEGG analyses showed primary enrichment in inflammatory responses (e.g., MAPK and PI3K-AKT pathways), cytokine activity (e.g., TNF signaling), and tumor necrosis factor-related pathways (Figure 7a,c). Curcumin significantly inhibited PI3K-AKT and Wnt signaling pathways (Figure 7d). Notably, 26 inflammation-related genes significantly upregulated by H_2_O_2_ were downregulated by curcumin intervention (comparing Figure 8a and Figure 7a).

**Figure 9:**
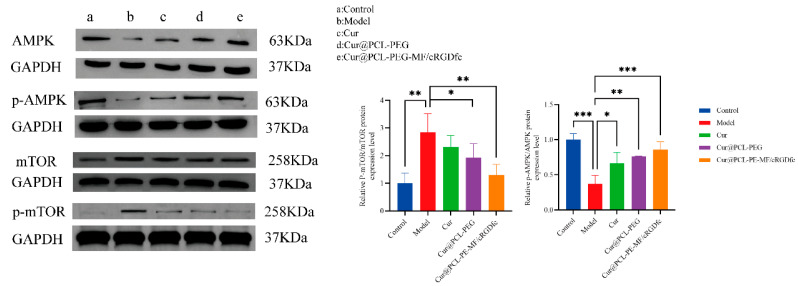
Curcumin and its nano-formulations modulate protein expression in the AMPK/mTOR pathway and inflammation-related pathways. Representative Western blots (**left panels**) show AMPK/mTOR pathway and inflammation-related proteins from three independent biological replicates, with GAPDH as the loading control. Quantitative analysis (**right panels**) presents mean ± SD of band intensities normalized to GAPDH (n = 3). Statistical significance was determined by one-way ANOVA followed by Tukey’s post hoc test. * *p* < 0.05, ** *p* < 0.01, *** *p* < 0.001.

Based on transcriptomic findings, related protein expression levels were examined. Drug treatments decreased Caspase-1 and GSDMD-N protein expression levels. Concurrently, inflammasome protein NLRP3 and inflammatory cytokines IL-1β and IL-18 in this pathway were downregulated to varying degrees, suggesting NLRP3/Caspase-1/GSDMD pathway activation alleviates cellular pyroptosis. Additionally, AMPK expression upregulation and mTOR expression downregulation indicated involvement of the AMPK/mTOR signaling pathway (Figure 8 and Figure 9).

In conclusion, oxidative stress disrupts AMPK signaling balance in ARPE-19 cells, impairing inflammatory response regulation and promoting downstream inflammatory cascade amplification, ultimately triggering pyroptosis. As a crucial cellular energy metabolism and stress response receptor, AMPK activity downregulation represents the key connection between oxidative stress and inflammatory amplification. Curcumin’s protective effects on ARPE-19 cells are primarily mediated through upstream AMPK signaling pathway activation, subsequently inhibiting abnormal downstream inflammatory signal activation, reducing pyroptosis executor molecule expression, and ultimately alleviating oxidative stress-induced cellular damage.

## 4. Discussion

In drug delivery carrier construction, we synthesized PCL-PEG-MF and PCL-PEG-cRGDfc, respectively. Their self-assembled nanoparticles under specific conditions effectively encapsulate poorly soluble components like curcumin, forming structures enabling sustained in vivo release. For PCL-PEG-MF modification, we employed the classic carbodiimide (EDC)-mediated condensation reaction, offering mild conditions, high efficiency, and strong specificity. This strategy specifically activates carrier terminal carboxyl groups, forming stable amide bonds with drug molecule amino groups. FTIR provided direct evidence for successful chemical bond formation. Compared to the blank carrier PCL-PEG-COOH spectrum, the PCL-PEG-MF spectrum showed characteristic amide bond absorption peaks, clearly indicating MF covalent bonding to the polymer backbone. Quantitative assessment via organic elemental analysis showed a measured nitrogen content consistent with theoretical stoichiometric calculation, confirming FTIR conclusions and verifying coupling reaction efficiency, ensuring expected stoichiometric drug loading. For PCL-PEG-cRGDfc synthesis, we utilized the highly efficient maleimide-thiol coupling reaction, widely applied in bioconjugation for fast reaction rate, high yield, and compatibility with aqueous/organic phases and particularly suitable for cysteine-containing peptide modification. ^1^H NMR characterization showed the PCL-PEG-cRGDfc spectrum exhibited broad peaks in the 7.0–8.5 ppm chemical shift range, attributed to cRGDfc cyclic peptide amide bond protons, absent in the PCL-PEG-MAL carrier spectrum, confirming successful cRGDfc connection. GPC analysis showed a molecular weight increase, indicating successful cRGDfc conjugation. FTIR results further confirmed cRGDfc modification success. Through two distinct yet equally efficient chemical coupling strategies, we precisely modified MF and cRGDfc on the PCL-PEG copolymer. cRGDfx targeting moiety specifically recognizes overexpressed integrin receptor αvβ3 on retinal pigment epithelial cells, achieving retinal region nanoparticle enrichment. MF targeting moiety enhances internalized nanoparticle affinity with mitochondrial complex I, regulating mitochondrial dysfunction and consequently modulating the AMPK/mTOR pathway.

Notably, previous studies have validated the feasibility of cRGD-targeted nanoplatforms for AMD therapy using classic retinal cell and animal models, which strongly supports the rationality of our targeting design. A prior study reported cRGD-functionalized nanoparticles for AMD treatment and verified their retinal protective effects based on ARPE-19 cells and rabbit CNV models, providing fundamental evidence for integrin-targeted retinal drug delivery [34]. Different from the single-targeted nanocarrier in that work, our system innovatively integrates dual cRGDfc-retinal targeting and MF-mitochondrial targeting, which can simultaneously improve retinal accumulation and intracellular mitochondrial regulation, thereby offering multi-level protection against oxidative stress-induced RPE injury, which may support multi-modal therapeutic intervention in AMD. In addition, a 2024 study focused on curcumin-metformin co-delivery nanoparticles and confirmed the synergistic therapeutic potential of these two bioactive molecules [35]. Compared with the previously reported co-delivery systems, our PCL-PEG-based dual-modified nanoparticles possess superior structural stability and sustained-release performance, which can prolong the retention time of curcumin and MF in retinal tissues and further optimize the synergistic protective effects against oxidative stress-induced RPE injury, which may contribute to improved anti-AMD therapeutic outcomes.

In evaluating curcumin and its targeted nano-formulations for AMD treatment, we adopted an in vitro assessment approach, primarily due to AMD pathological complexity and retinal anatomical challenges [36], which make direct in vivo studies difficult for precise mechanism and efficacy analysis. Following in vitro pharmacodynamic evaluation, we will pursue in vivo experiments or organoid models. We selected the H_2_O_2_-induced retinal injury model because oxidative stress plays a key role in AMD pathogenesis, and H_2_O_2_ as a common oxidative stress inducer effectively simulates the oxidative damage environment RPE cells experience in AMD, facilitating curcumin-targeted nanoparticle antioxidative stress potential assessment. We selected ARPE-19 and HUVEC as in vitro models because wAMD lesions primarily localize in the retina, and ARPE-19 cells derived from adult retinal pigment epithelium are commonly used in wAMD-related experiments, reflecting drug effects on the retina [37,38]. HUVECs simulate vascular endothelial cell behavior, evaluating drug anti-angiogenic effects crucial for choroidal neovascularization study in wet AMD. These cell models represent key AMD pathogenesis aspects—oxidative stress damage and neovascularization—providing a scientific basis for evaluating curcumin-targeted nanoparticle multifaceted pharmacological activities. To explore the potential mechanisms underlying curcumin-mediated protection against AMD-related injury, we first performed RNA-seq analysis on cells treated with free curcumin. This transcriptomic screening identified the AMPK/mTOR and NLRP3 pathways as significantly modulated signaling cascades, guiding our subsequent mechanistic validation. It should be noted that the RNA-seq experiment was conducted only on the free curcumin group, and the pathway identification is based on these data. Subsequent Western blot validation, however, included both the free curcumin group and the Cur@PCL-PEG-MF/cRGDfc nanoformulation group, confirming that both formulations regulated these two key pathways in a consistent manner. Thus, the pathway conclusions are supported by transcriptomic screening with free curcumin and cross-formulation validation using Western blot analysis.

While in vitro models cannot fully replicate the complex in vivo microenvironment, cell–cell interactions, and drug metabolism processes, the current experiments yield promising results demonstrating curcumin-targeted nanoparticles’ potential against age-related macular degeneration (AMD) at a cellular level. These findings establish a foundation for subsequent in vivo experiments verifying nanoparticle efficacy and safety in animal models, which are essential for clinical translation. Furthermore, thorough investigation of curcumin nanoparticles’ long-term toxicity, biodistribution, and pharmacokinetic characteristics represents a crucial step toward clinical application. Comprehensive understanding enables formulation optimization, ensuring high efficacy and limited side effects. Importantly, recent clinical and preclinical safety research on metformin application in advanced AMD provides essential guidance for our subsequent in vivo safety evaluation. A recent study pointed out that metformin may induce retinal fibrosis and exacerbate pathological progression in late-stage nAMD, reminding us of the potential stage-dependent safety risks of metformin-based therapy [39]. Although our current in vitro results demonstrated that MF-modified nanoparticles could effectively repair mitochondrial dysfunction and alleviate oxidative damage without adverse effects, the pro-fibrotic risk of metformin in advanced AMD cannot be ignored. Therefore, our follow-up in vivo experiments will focus on detecting retinal fibrosis-related indicators, especially in advanced AMD models, to systematically verify the biosafety of our curcumin/MF co-loaded targeted nanoparticles and avoid potential adverse reactions. The present study does not include experiments to evaluate the individual contributions of MF or cRGDfc alone to pharmacodynamics, mechanisms, or the separate roles of targeting versus cell penetration; these aspects will be explored in future work.

## 5. Conclusions

This study prepared biocompatible carrier material PCL-PEG via organic synthesis, subsequently constructing PCL-PEG-MF and PCL-PEG-cRGDfc through amidation and Michael addition reactions. These were mixed with curcumin to prepare Cur@PCL-PEG-MF/cRGDfc nanoparticles. Structural evaluation using FTIR, ^1^H NMR, XRD, EDS, and organic elemental analysis confirmed nanoparticle and carrier structures. Nanoparticles exhibited small size, storage stability, and enhanced affinity for oxidative stress-experiencing ARPE-19 cells, achieving near-100% cellular uptake. Following internalization, they alleviated oxidative stress damage, reduced ROS and LDH release, decreased DNA fragmentation, and lowered VEGF-A expression levels, thereby inhibiting angiogenesis. Transcriptomic studies suggested curcumin and its nano-delivery systems may exert protective effects by influencing the AMPK/mTOR pathway and subsequently modulating downstream NLRP3/Caspase-1/GSDMD signaling. Subsequent experiments verified that curcumin and its nano-formulations decrease inflammatory factor expression (IL-1β, IL-18), reduce Caspase-1, GSDMD-N, and NLRP3 pathway protein levels, and increase AMPK protein expression, activating the AMPK pathway. These results demonstrate successful development of a curcumin nano-formulation with retinal targeting, antioxidative stress, and anti-angiogenic properties, establishing a foundation for further in vivo efficacy and pharmacokinetic studies while providing a novel formulation option for AMD treatment.

## Data Availability

The original contributions presented in this study are included in the article. Further inquiries can be directed to the corresponding authors.
